# *cagA* gene EPIYA motif genetic characterization from Colombian *Helicobacter pylori* isolates: Standardization of a molecular test for rapid clinical laboratory detection

**DOI:** 10.1371/journal.pone.0227275

**Published:** 2020-01-10

**Authors:** Eliana Rocío Rodríguez Gómez, William Otero Regino, Pedro A. Monterrey, Alba Alicia Trespalacios Rangel

**Affiliations:** 1 Department of Microbiology, Pontificia Universidad Javeriana, Bogotá, Colombia; 2 Gastroenterology Unit, Clínica Fundadores, Bogotá, Colombia; 3 School of Natural Sciences and Mathematics, Universidad del Rosario, Bogotá, Colombia; Mercy Hospital, SIERRA LEONE

## Abstract

The aim of this work was to determine current *cagA* gene EPIYA motifs present in Colombian *Helicobacter pylori* isolates using a fast and reliable molecular test. DNA from eighty-five *Helicobacter pylori-cagA* positive strains were analyzed. Strains were obtained from patients diagnosed with functional dyspepsia at Clínica Fundadores in Bogotá. The 3' region of the *cagA* gene was amplified through conventional Polymerase Chain Reaction (PCR). Obtained amplicons were sequenced using the Sanger method and analyzed with bioinformatics tools. Additionally, a significant Spearman correlation coefficient was determined between the patients' age and the number of EPIYA-C repeats; with *p* values < 0.05 considered significant. Estimates were obtained using a 95% CI. The 3´ variable region of the *cagA* gene was amplified and PCR products of the following sizes corresponded to the following EPIYA motifs: 400 bp: EPIYA AB, 500 bp: EPIYA ABC, 600 bp: EPIYA ABCC and 700 bp: ABCCC. A single PCR band was observed for 58 out of 85 *Helicobacter pylori* isolates, with an EPIYA distribution motif as follows: 7/85 AB (8.2%), 34/85 ABC (40%), 26/85 ABCC (30.6%) and 18/85 ABCCC (21.2%). However, in 27 out of 85 *Helicobacter pylori* isolates, two or more bands were observed, where the most predominant *cagA* genotype were ABC-ABCC (26%, 7/27) and ABCC-ABCCC (22.2%, 6/27). A direct proportionality between the number of EPIYA-C repeats and an increase in the patients’ age was observed, finding a greater number of EPIYA ABCC and ABCCC repeats in the population over 50 years old. All isolates were of the Western *cagA* type and 51.8% of them were found to have multiple EPIYA-C repeats. These standardized molecular test allowed to identify the number of EPIYA C motifs based on band size.

## Introduction

*Helicobacter pylori (H*. *pylori)* bacteria infecting human gastric mucosa has been associated with development of gastroduodenal diseases, such as chronic gastritis, peptic and duodenal ulcer, MALT lymphoma and gastric cancer. Some populations displaying high infection prevalence, also present a high incidence of gastric cancer (East Asia). In contrast, this same relationship does not apply to Africa and South Asia. A plausible explanation for these differences might be due to *H*. *pylori* genetic plasticity, suggesting geographical differences in virulence genes [1]. However, gastric cancer is a multifactorial disease, where genetic and environmental factors, as well as *H*. *pylori* infection are involved. Some of the characteristics attributed to this microorganism are virulence factors, where the most studied and important is the CagA protein [2].

*cagA* gene encodes a highly immunogenic and polymorphic protein (CagA). The protein is delivered into the gastric epithelial cells through the type IV secretion system. Once released within the cell, CagA is phosphorylated by the Src kinases family in EPIYA tyrosine residue motifs (Glu-Pro-Ile-Tyr-Ala), located in the carboxyl-terminal region [3].

After phosphorylation, CagA interacts with host proteins involved in signaling pathways, cell growth regulation, morphogenesis, cell motility and polarity, such as phosphatase SHP-2, an oncoprotein which has recently been found in a variety of human malignancies. The CagA-SHP-2 interaction activates the Erk MAP kinase cascade in dependent and independent ways, eliciting an epithelial cell morphological transformation, which leads to cell elongation (also known as the “Hummingbird phenotype”) [4]. In addition, Erk activation in epithelial cells also actives NF-Kb, which in turn induces interleukin-8 (IL-8) expression [5].

On the other hand, sustained SHP-2 activation induces apoptosis in gastric epithelial cells. However, CagA protein binding to the CSK kinase carboxyl-terminal region brings about down regulation, resulting in a CagA-SHP-2 complex decrease, contributing to dephosphorylation of other SRC substrate proteins, such as cortactin, a cytoskeletal protein that may be involved in morphological changes induced by CagA [6].

CagA variation in size is due to the EPIYA motif. Additionally, based on the number of amino acids and the flanking sequence of the motif, four types of EPIYA motifs have been described (A, B, C and D) [7]. A and B motifs are present in all isolates, whereas motif C is present in Western strains and motif D in Asian strains. EPIYA C motif presents different repeats, which are related with tyrosine phosphorylation levels. Therefore, Western CagA strains that have the highest EPIYA-C repeats are biologically more active than those with fewer repeats [8]. Accordingly, five studies have been conducted in order to assess the risk of developing gastric cancer based on the number of EPIYA-C repeats. Two of these studies were conducted in Europe [9,10]. Based on the presence of two or more EPIYA-C repeats both studies reported the risk of developing gastric cancer was increased by 32 and 51-fold, respectively. The other three studies were conducted in America (Mexico) [11], (Brazil) [12] and (Colombia)[13], reporting the risks (OR) of developing gastric cancer increased by 5.9, 3.8 and 12-fold with the presence of the following repeats: 2 EPIYA-C, 2 and 3 EPIYA-C and 3-EPIYA-C, respectively. Additionally, to evaluate the association between EPIYA-C motif and gastric cancer risk a meta-analysis was performed. It was reported multiple EPIYA-C phosphorylation sites were associated with increased gastric cancer (OR: 2,95, 95%, *P* < .001). A subgroup analysis of different regions was performed finding in the South America, North American and Europe a significant association of multiple EPIYA-C motifs with cancer gastric (OR = 3.06, 95% CI: 2.29–4.08, *P*<0.001; North America: OR = 4.59, 95% CI: 1.32–15.94, *P* = 0.017; Europe: OR = 3.69, 95% CI: 1.98–6.88, *P*<0.001 [14].

In contrast to EPIYA-C motif, EPIYA-D motif rarely duplicates; however, EPIYA-D consensus sequence exhibits greater SHP-2 binding activity, since there are no changes in amino acid sequences. On the other hand, EPIYA-C motif presents an amino acid sequence change at position pY+5. Therefore, EPIYA-D motif presents a stronger ability to induce stark changes in epithelial cells and a higher morphogenetic activity, when compared to EPIYA-C motif. Consequently CagA Asian strains tend to be more carcinogenic than CagA Western strains [6].

There are few studies that have described *cagA* gene EPIYA motifs genetic diversity present in *H*. *pylori* isolates from patients infected with chronic gastritis in Colombia. This information is very important to define *H*. *pylori* genetic diversity for this gene. Moreover, further studies could shed light on how these genetic differences and EPIYA motifs are related to gastric cancer onset. Current publications have focused on this diversity only in patients with gastric cancer. Hence, this information does not reflect the existing circulating diversity, which allows development of additional studies to assess the impact of *cagA* gene diversity on gastroduodenal diseases. To contribute with this understanding, this study aimed to determine current *cagA* gene EPIYA motifs circulation in Colombian *H*. *pylori* isolates through a rapid molecular test. In addition, the number of EPIYA motifs were classified according to the patients’ age to provide information regarding bacteria virulence and its possible relationship with gastric disease development.

Additionally, according to amplicon molecular weight this study allowed to determine the number of EPIYA motifs present in the *cagA* gene. This information will be very useful for gastroenterologists and clinicians to diagnose a patient by using accurate *cagA* gene information, thus determining if a patient infected with the bacteria could be at risk of developing gastric cancer. Furthermore, this technique spares the need of sequencing, making it a useful tool for clinicians to implement and confirm *cagA* infection phenotype.

## Materials and methods

### *H*. *pylori* isolates

dx.doi.org/10.17504/protocols.io.54jg8un

85 *H*. *pylori* strains *cagA* gene positive from Bogotá, Colombia were examined. *H*. *pylori* clinical isolates were obtained from patients diagnosed with functional dyspepsia at Clínica Fundadores in Bogotá, between 2009 and 2010. This study was approved by the Institutional Ethics Committees from Universidad Javeriana and Clínica Fundadores with signed informed consent from all patients.

Isolates were molecularly characterized and previously genotyped by our research group and were stored in the Collection of microorganisms at Pontificia Universidad Javeriana (CMPUJ) in Bogotá, Colombia.

### PCR amplification and sequencing

The entire 3´ repeat region for c*agA* gene was amplified by polymerase chain reaction (PCR) using *cagA* primers, sense (CAGTF 5’-ACCCTAGTCGGTAATGGG-3’) and antisense primers (CAGTR 5’ GCTTTAGCTTCTGAYACYGC 3’, Y: C + T) position 2536 to 3044 [15] (GenBank accession number L11714). *H*. *pylori* reference strain (NCTC 11637, *cagA*-positive) was used as a positive control and *H*. *pylori* strain negative for *cagA* gene, *Staphylococcus aureus*, *Salmonella typhi* and *Escherichia coli* strains were used as a negative controls.

PCR amplification was carried out in a volume of 50 μL containing 0.3 μM primer concentration (Invitrogen, Carlsbad, CA, USA), 100 ng *H*. *pylori* genomic DNA and 1 U *Taq* DNA polymerase master mix (Promega, Madison, USA).

Polymerase chain reaction conditions included an initial denaturation step at 95°C/10 min, followed by 39 cycles (95°C/30 sec, 52.3°C/30 sec and 72°C/ 36 sec) and a final extension cycle at 72°C/5 min. PCR amplification was carried out in a Thermal cycler T100 (Biorad, Hercules, CA, USA). PCR products were visualized in 2% agarose gel electrophoresis with Syber Safe and photographed in Gel Doc^XR+^ system (Biorad, Hercules, CA, USA) for analysis.

Samples which presented two or more bands in agarose gels from PCR amplifications (samples with mixed infection), were visualized using an UV transilluminator and cut. Each gel band with different sizes was placed in an Eppendorf tube and purified using Wizard SV Gel and PCR Clean-up System Kit (Promega, Madison, USA), according to the manufacturer’s instructions. DNA concentration was determined by spectrophotometry using NanoDrop 2000 (Thermo Scientific, Wilmington, NC). DNA amplification obtained from gel bands was subsequently amplified by conventional PCR. Sequencing for PCR products and bioinformatic analysis were carried out to determine presence of EPIYA motifs in each sample, and to establish different strain types infecting each patient.

All PCR products from this study were sequenced at Universidad de Los Andes, Bogotá, Colombia. Sequencing reactions were carried out for both DNA strands (forward and reverse sense) by the Sanger method using as control *H*. *pylori* reference strain (NCTC 11637) sequence, which is Western *cagA* strain containing the EPIYA-ABC motif.

### Bioinformatic analysis for sequences containing the EPIYA motif

Nucleotide sequences were aligned and analyzed using BLASTx, including *H*. *pylori* reference strain *cagA* sequences (NCTC 11637) (GenBank access AF202973).

The deduced peptide sequences containing EPIYA motifs were aligned employing CLUSTAL W (European Bioinformatics Institute http://www.ebi.ac.uk/Tools/clustalw2/).

### Statistical analysis

Spearman correlation coefficient was calculated between the patients’ age and the number of EPIYA-C repeats, using a *p* value of less than 0.05 to establish significance. Estimation was obtained using a 95% CI, with a Bootstrap with 1,000 repetitions. The statistical processing was carried out in STATA 14.

## Results

https://dataverse.harvard.edu/dataset.xhtml?persistentId=doi:10.7910/DVN/KCZNKP

### PCR amplification of *cagA* gene variable 3’ region

*cagA* gene variable 3’ region coding for EPIYA motifs in 85 *H*. *pylori* isolates was amplified by using PCR. Products ranged in sizes between 400 bp and 700 bp. Based on this sizes, seven isolates displayed fragments of 400 bp, 34 isolates of 500 bp, 26 isolates of 600 bp and 18 isolates of 700 bp. Products were visualized in agarose gel electrophoresis. It was observed amplicons were equidistantly organized (100 bp) in a ladder-like arrangement, evidencing *cagA* gene 3´ region size variation was due to presence of multiple EPIYA repeated sequences. DNA from the *H*. *pylori* reference strain (NCTC 11637) exhibited a single band with a size of 500 bp.

In 58 out of 85 (68.2%) *H*. *pylori* isolates, a single-band amplicon was observed, where PCR products ranged between 400 bp and 700 bp (Table 1). However, in 27 out of 85 isolates (31.8%) two or more bands were present. Most of the isolates presented two bands with sizes ranging from 500, 600 and 700 bp (Table 2).

**Table 1:** PCR product sizes of isolates with single-band.

Product size (bp)	Number of isolates
400	2
500	24
600	18
700	14

**Table 2:** PCR products of isolates with two or more bands.

Number of isolates	Number of bands	Product size (bp)
7	2	500–600
6	2	600–700
5	2	400–500
5	3	400-500-600
1	3	500-600-700
1	3	400-500-700
2	4	400-500-600-700

### EPIYA motif bioinformatic analysis in *H*. *pylori* clinical isolates

**Fig 1:**
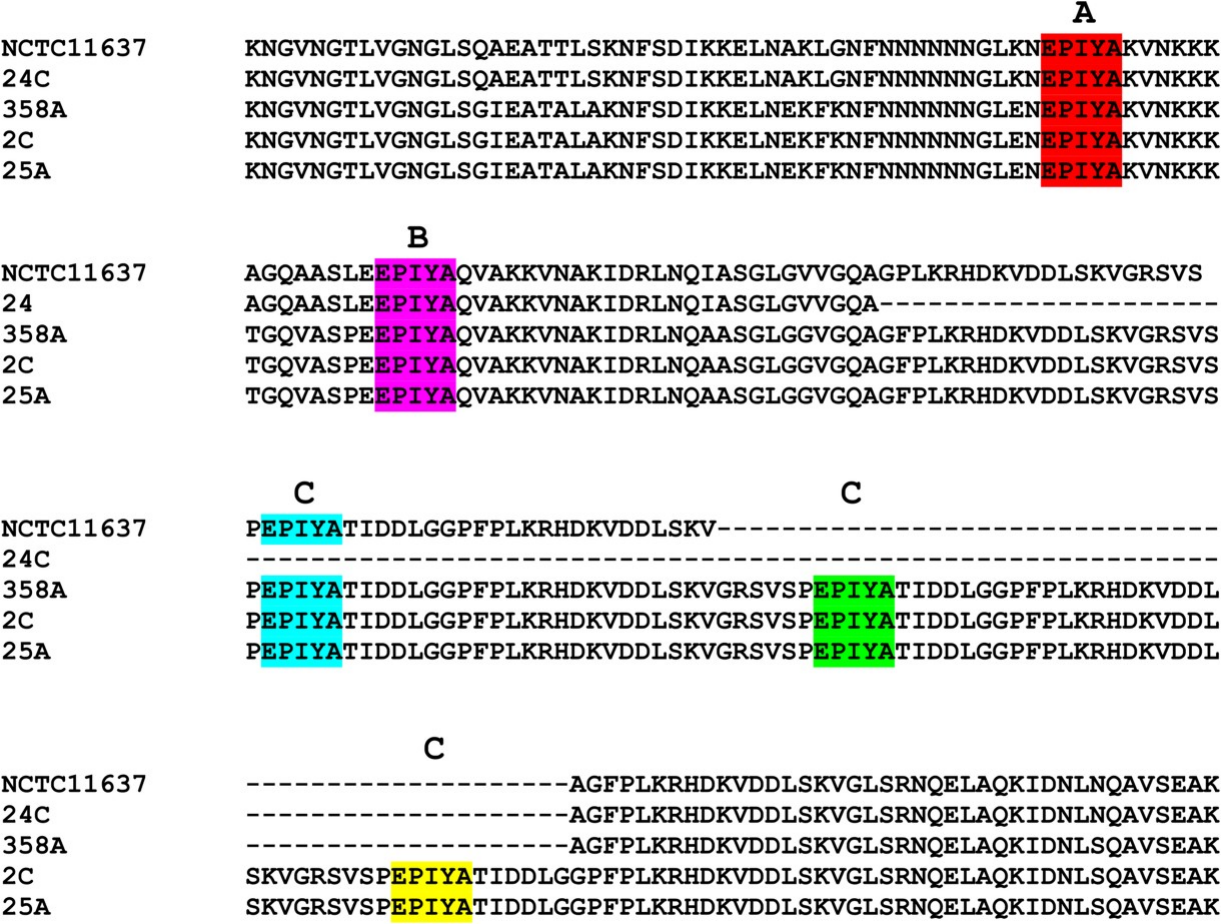
3´*cagA* region amino acid sequence alignment for five Colombian *H*. *pylori* isolates. EPIYA motifs: A (red), B (purple), one C (blue), two C (green), three C (yellow). *H*. *pylori* reference strain (NCTC 11637) contained the EPIYA-ABC motif, strains 24C (AB), strain 358A (ABCC), strains 2C and 25A (ABCCC).

**Fig 2:**
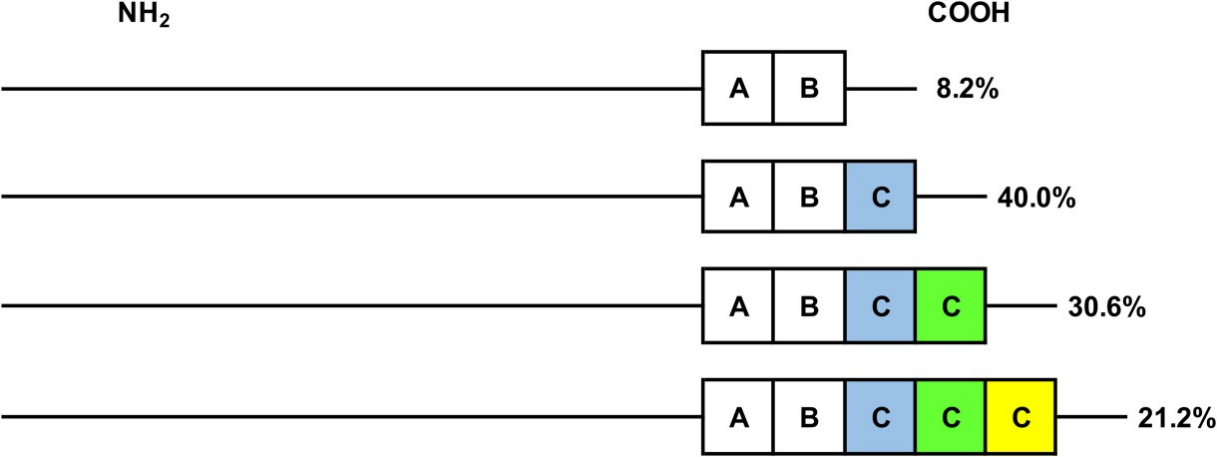
Total distribution of EPIYA motif in Colombian *H*. *pylori* isolates. EPIYA-motif in the CagA protein C-terminal region and their frequency in Colombian *H*. *pylori* isolates included in this study. The EPIYA motif with one C (blue), two Cs (green) and three Cs (yellow) are shown.

Based on CLUSTAL W peptide sequence alignment it was observed all sequenced *cagA* variable regions corresponded to the Western type, with three EPIYA motifs: EPIYA-A: EPIYAKVNKKKAGQ; EPIYA-B: EPIY(A/T)QVAKKVNAKI and EPIYA-C: EPIYATIDDLGGP. From the population studied no strain corresponded to the EPIYA-D Eastern type: EPIYATIDFDEANQAG (Fig 1). Total distribution of EPIYA motif in isolates was: 7/85 AB (8.2%), 34/85 ABC (40%), 26/85 ABCC (30.6%) and 18/85 ABCCC (21.2%) (Fig 2).

### Analysis of the *cagA* EPIYA-C repeat number according to band size by rapid molecular test

**Fig 3:**
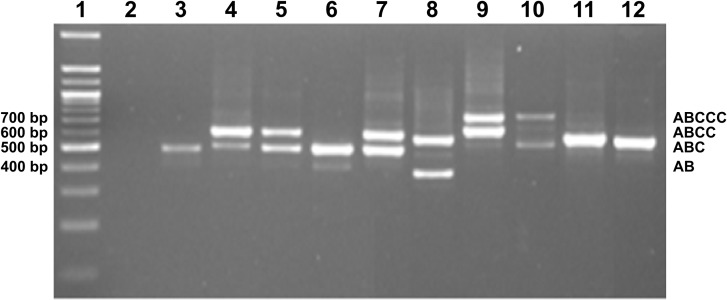
Association between PCR amplicons size and EPIYA motif. DNA from *H*. *pylori* isolates (lanes 3–12) were amplified and PCR products were visualized in a 2% agarose gel. The following PCR products are illustrated: 400 bp (EPIYA AB), 500 bp (EPIYA ABC), 600 bp (EPIYA ABCC) and 700 bp (EPIYA ABCCC). Lane 2: negative control, lane 12: NCTC 11637, Lane 1: 100 bp DNA ladder (Promega, USA).

EPIYA C repeat number prediction based on PCR amplicon size was confirmed by sequencing. To this end, first standardization of conventional PCR was carried-out to amplify the fragment containing EPIYA motifs. Following, each band obtained in the PCR process was cut and sequenced, identifying that all 400 bp PCR products corresponded to EPIYA AB, 500 bp to EPIYA ABC, 600 bp to EPIYA ABCC and 700 bp to EPIYA ABCCC (Fig 3), with the following distribution: 7/85 isolates with 400 bp product size were EPIYA AB, 34/85 500 bp isolates were EPIYA-ABC, 26/85 isolates with 600 bp products were EPIYA-ABCC and 18/85 isolates with 700 bp were EPIYA-ABCCC (Table 3).

**Table 3:** PCR amplicons size and EPIYA motif distribution.

Product size (bp)	EPIYA motif	Number of isolates
400	AB	7
500	ABC	34
600	ABCC	26
700	ABCCC	18

Furthermore, presence of single and multiple *cagA* EPIYA genotypes was observed in patients included in this study. In 58 patients no co-infection was identified and the following EPIYA distribution was observed: 2/58 had the AB motif (3.5%), 24/58 had the ABC (41.4%), 18/58 had the ABCC (31%) and 14/58 had the ABCCC (24.1%) (Table 4). 27 patients were infected with two or more different *cagA* EPIYA genotypes. Co-infection with two EPIYA genotypes was detected as follows: ABC and ABCC were observed in 7 patients; ABCC and ABCCC in 6 patients and AB and ABC in 5 patients. Additionally, co-infection with three and four EPIYA genotypes was also observed. Concomitant genotypes with three different motifs were observed as follows: AB, ABC and ABCC in five patients; AB, ABC and ABCCC in one patient; ABC, ABCC and ABCCC in one patient and last, two patients had coinfection with strains with the following EPIYA genotypes: AB, ABC, ABCC and ABCCC (Table 5).

**Table 4:** *cagA* EPIYA genotypes in patient isolates without mixed infection.

Number of isolates	EPIYA motif type	EPIYA motif %
2	AB	3.5
24	ABC	41.4
18	ABCC	31
14	ABCCC	24.1

**Table 5:** *cagA* EPIYA genotypes in patient isolates with coinfection.

Number of isolates	EPIYA motif combination	EPIYA motif combination %
5	AB-ABC	18.5
7	ABC-ABCC	26
6	ABCC-ABCCC	22.2
5	AB-ABC-ABCC	18.5
1	AB-ABC-ABCCC	3.7
1	AB-ABCC-ABCCC	3.7
2	AB-ABC-ABCC-ABCCC	7.4

### Correlation between EPIYA-C repeats and patients’ age

**Fig 4:**
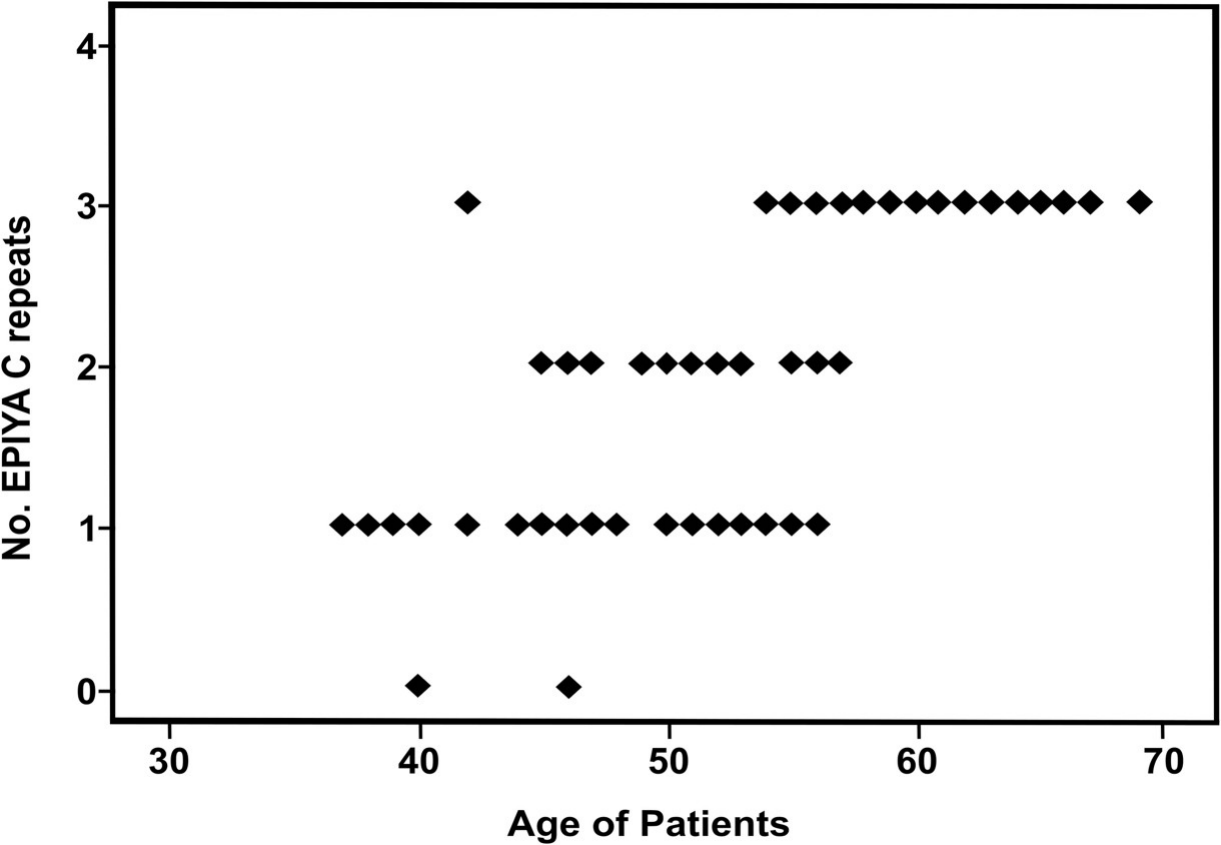
Correlation between patients’ age and the number of EPIYA-C repeats. Total number of EPIYA C repeats in 85 isolates from patients infected with *H*. *pylori* and patients’ age.

For the 85 isolates Spearman correlation coefficient between the patients’ age and the total number of EPIYA-C repeats was 0.822, suggesting a direct proportionality between the number of EPIYA-C repeats and an increase in the patients’ age (Fig 4). The following was concluded from the statistical analysis: patients without coinfection with one EPIYA-C repeat on average were 45 years old, two EPIYA-C repeats had on an average 51 years, and with three EPIYA-C repeats on average had an age of 63 years. A significant correlation was observed between increase in the patients’ age and the number of EPIYA-C repeats (*P* < 0.05).

## Discussion

All isolates included in this study were of the Western *cagA* gene type. No Eastern type isolates circulating within our population were found, which is agreement with studies also included Colombian patient isolates [15–17]. Furthermore, studies in Europe, America and Africa also reported the Western *cagA* gene type [9,18–20].

Presence of *cagA* gene with one to three EPIYA-C repeats from *H*. *pylori* isolates were observed, which were found to contain EPIYA ABC segments in 40% of *H*. *pylori* isolates (Fig 2). These results are similar the one reported in other studies [12,13,15,21]. However, the frequency of isolates containing a *cagA* gene with two or three EPIYA-C repeats was 51.8%, a higher percentage than the one reported in studies from Colombia [13,16,17,21,22]. Differences might be accounted by the fact that previous studies included patients with different diseases (atrophic gastritis, ulcers, cancer), unlike the present study where circulation of isolates with multiple EPIYA-C was described only from patients with chronic gastritis.

Likewise, to determine the prevalence of isolates with multiple EPIYA-C motifs in other countries, different studies have been carried-out. Fewer isolates with multiple EPIYA-C have been reported in France (32%)[22], Costa Rica (30.3%)[23], South Africa (27%)[20], Greece (21%) [24], Cuba (22.1%) [25], Mexico (20%) [26], USA (17%) [22] and Peru (11.5%) [19] in comparison with those found in this study. This might be due to the process of *cagA* microevolution, which describes a genetic change that leads to a modification in virulence factors, representing important implications in *H*. *pylori* pathogenesis [27,28]. This might explain in part the greater number of EPIYA-C in patients with chronic gastritis in this study, but the relationship between bacterial virulence and disease did not show a strict association. Additionally, another possible cause for results observed in this study is the populations studied. Previous studies included patients with different gastrointestinal diseases (gastritis, ulcers, metaplasia, dysplasia, cancer) with a variation in the sample size and possibly, gastric cancer incidence for each country. It is noteworthy that Colombia has a higher incidence of gastric cancer in comparison with other countries, possibly due in part to multiple EPIYA-C repeats. Western CagA proteins with two or more EPIYA-C repeats are more virulent and associated with a high gastric cancer risk than isolates with only one EPIYA-C motif [9,21,25]. Moreover, the risk of gastric cancer is 12 times higher in patients infected with three EPIYA-C repeats than in patients infected with one EPIYA-C motif [13].

The results of this study showed a significant association between the increase in patients’ age and the EPIYA repeats observed (Fig 4). Gastric cancer is uniformly rare in adults aged <50 years and frequently occurs in an age range between 55–80 years, in younger patients it is unusual [29]. Likewise, the International Agency for Research on Cancer (IARC) and World Health Organization (WHO) (2008) reported the mortality of gastric cancer increased with advancing age. They reported 245 deaths occur in patients between the ages of 40–49 years old, 479 deaths in patients aged between 50–59 years old and 687 deaths in patients aged 60–69 years old. Thus, suggesting in the present study patients between 50–70 years old, who presented two and three EPIYA-C repeats, might be at increased risk of developing gastric cancer rather than under 50 years who had one or no EPIYA C motif. These results agree with those reported in Iraqi patient isolates [30], where the average age of patients infected with EPIYA ABCC and ABCCC was higher than in patients who had other types of EPIYA motifs, finding the following EPIYA distribution related to the average age in patients: EPIYA ABC- 45 years, EPIYA ABCC- 51 years and EPIYA ABCCC- 63 years. However, they included different pathologies, not only chronic gastritis.

According to estimates from the International Agency for Research on Cancer (IARC) GLOBOCAN project, worldwide there were 1,033,701 new cases of gastric cancer and 782,685 deaths related to gastric cancer in 2018 [31]. Additionally, about 40% of patients with gastric cancer do not report symptoms before diagnosis, prevention becomes the best strategy to control gastric cancer. A program to prevent gastric cancer should include: detecting *H*. *pylori*, surveillance of precancerous lesions by endoscopic and histological studies, improve sanitation and hygiene, restrict salt consumption in the diet, and eat a balanced and rich diet containing fruits and fresh vegetables, with plenty of antioxidants [32]. Since more than 80% of cases of gastric cancer have been attributed to *H*. *pylori* infection [33], eradication is considered a primary prevention strategy.

According to the Maastricht V consensus [34], *H*. *pylori* eradication reduces the risk of developing gastric cancer. Therefore, patients should be treated during the initial phase of infection, before pre-neoplastic changes develop in the gastric mucosa. Furthermore, to reduce the risk of gastric cancer consensus statements suggested an eradication therapy should be offered to family members that have a first consanguinity degree of gastric cancer patients, patients with previous gastric neoplastic that have already been treated by endoscopic or gastric resection subtotal, patients with a risk of severe pangastritis, corpus predominant gastritis or severe atrophy and patients with chronic inhibition of gastric acid for more than one year. Two other documents, one written by the American College of Gastroenterology and the other by the Asia and Pacific consensus, also recommended *H*. *pylori* eradication in patients with endoscopic resection of early gastric cancer in regions with annual incidence of gastric cancer with a population above 20/100,000 [35].

In this study, 27 isolates with two or more bands with sizes corresponding to different numbers of EPIYA-C repeats were found (Table 2). This is probably due to sub-clone presence with high rates of plastic recombination with *H*. *pylori* genome, emerging from microevolution processes or to co-infection with multiple *H*. *pylori* strains that coexist in the same patient [36], also may be due to that *H*. *pylori* isolates from gastric biopsies is often made by the sweeping method instead of individual colony selection [24]; hence, presence of multiple *cagA* genotypes in the same patient was observed.

With respect to the methodological strategy used in this study, PCR amplification allowed to establish the number of EPIYA repeats, according to PCR product size and number of bands (Fig 3), which was further confirmed by sequencing. This method permitted to predict with precision each of the bands specific for *cagA* sequences, in addition to the presence of co-infection with different *cagA* genotypes. Therefore, its implementation can be very useful in hospital laboratories. Furthermore, this technique does not require sequencing, and represents a useful tool for clinical implementation, thus helping to confirm CagA phenotype infection.

Other methodologies, such as design specific primers for Western *cagA*, Oriental *cagA*, EPIYA-A, B and C repeats and sequencing have been previously reported [18,25]. These strategies also detect strains with more than one EPIYA motif in the same patient, namely mixed infection or co-infection but the disadvantage of these methods include a longer processing time and higher costs.

## Conclusion

In this study, it was identified that all Colombian *H*. *pylori* isolates contained the Western *cagA* gene type, with a predominant EPIYA-ABC and multiple EPIYA-C repeats. EPIYA-ABCC and EPIYA-ABCCC were present in 52% of the infected population, indicating that *H*. *pylori cagA* positive isolates have a high virulence potential and therefore, these strains could be involved in a higher risk of gastric cancer development. In the population over 50 years old a greater number of EPIYA ABCC and ABCCC motifs was observed, moreover mixed infection with strains with different EPIYA C was frequent. Taking into account that the objective of our study was to determine current c*agA* gen circulation present in EPIYA motifs, justifying why only genomics was performed in this study. EPIYA motifs genotype not necessarily imply a permanent expression. Therefore, we suggest for future studies to perform transcriptomics or carry-out an *in vivo* approximation at the mucosa level, assay translocation and tyrosine CagA phosphorylation, IL-8 secretion and cell elongation induction, which would allow us more comprehensively to understand the mixed infection with different EPIYA motif and clinical significance.

Standardized molecular biology tests in this study are easy to carry-out and according to the band size allow to determine the number of EPIYA C motifs repeats. This method can be easily implemented in hospitals and contribute with *cagA* genotyping in Colombia. Furthermore, it will be a useful tool for gastroenterologists to better assess patients infected with *H*. *pylori* and the risk of developing gastric cancer.
