# Correction: *cagA* gene EPIYA motif genetic characterization from Colombian *Helicobacter pylori* isolates: Standardization of a molecular test for rapid clinical laboratory detection

**DOI:** 10.1371/journal.pone.0228840

**Published:** 2020-02-03

**Authors:** Eliana Rocío Rodríguez Gómez, William Otero Regino, Pedro A. Monterrey, Alba Alicia Trespalacios Rangel

An additional affiliation is missing for the second author. William Otero Regino is also affiliated with Gastroenterology unit, Universidad Nacional de Colombia, Bogotá, Colombia.
